# Mechanism and Control of Black Spot Deterioration on Lacquered Architectural Components of Dajue Temple

**DOI:** 10.3390/microorganisms14051107

**Published:** 2026-05-13

**Authors:** Sifan Ai, Yu Wang, Jiao Pan, Gang Hu, Ruiting Zhao

**Affiliations:** 1 Capital Museum, Beijing 100045, China; m18222389396@163.com; 2 School of Archaeology and Museology, Peking University, Beijing 100871, China; 3 Institute for Cultural Heritage and History of Science & Technology, University of Science and Technology Beijing, Beijing 100083, China; d202310766@xs.ustb.edu.cn (Y.W.); jiaopan@ustb.edu.cn (J.P.)

**Keywords:** wooden cultural heritage, microbial management, cultural heritage conservation, fungicides

## Abstract

Dajue Temple, a representative ancient architectural heritage in North China, houses numerous lacquered wooden components of exceptional historical and artistic value. Prolonged environmental exposure causes severe dark discoloration and black spotting on lacquer surfaces, threatening their structural integrity. This first investigation into the damage identifies the spots as microbial in origin, with *Cladosporium* spp. as the primary agent driving deterioration and possessing wood-degrading capabilities. Antifungal tests show that thymol, clove essential oil, and nano-silver gel are all effective inhibitors. We proposed targeted, relic-friendly microbial control strategies tailored for ancient lacquered wooden components. These findings provided scientific guidance for the sustainable conservation and restoration of lacquered architectural elements in historic temples and comparable cultural heritage sites. In future work, environmental monitoring and the biocides’ compatibility should be involved, which will help to clarify microbe–environment interactions, enable early warning of biodeterioration risks and explore the wood-friendly biocides.

## 1. Introduction

Located in Haidian District, Beijing, Dajue Temple was initially built in the Tang Dynasty and renovated across successive dynasties. Serving as a prominent imperial prayer temple, it is renowned as a representative ancient monastery of the Western Hills. Retaining abundant Liao, Ming and Qing cultural relics and complete ancient architectural layouts, the temple is officially recognized as a National Key Cultural Relics Protection Unit of China. However, in recent years, a pervasive occurrence of black discoloration has been observed on the surfaces of painted components, including wooden columns and door and window frames within major halls such as the Mahavira Hall and the Qingyun Xuan. This esthetic degradation signals potential underlying material deterioration, thereby posing a significant threat to the long-term preservation of these structures [1,2].

Preliminary cleaning efforts revealed that the formation of these black stains is not attributable to superficial deposits such as smoke or settled dust, suggesting a possible involvement of microbial infestation. Should microbial deterioration be confirmed, the implications extend far beyond mere surface discoloration. The secretion of hydrolytic enzymes, including cellulase and laccase, along with various organic acids by microorganisms, can degrade the principal components of wood—cellulose, hemicellulose, and lignin—resulting in structural weakening, pulverization, and even perforation [3,4,5,6]. Critically, the observation of these black stains overlying the protective layers of lacquer and tung oil finish indicates that the causative microorganisms may have already breached the traditional physical and chemical barriers designed for conservation. Without timely intervention, sustained microbial metabolic activity could alter local microenvironments, facilitate deeper hyphal penetration, and ultimately lead to extensive lacquer exfoliation and exposure of the underlying wooden substrate, culminating in irreversible damage to these invaluable cultural artifacts.

Within the discipline of cultural heritage conservation, a consensus emerged during the 1980s identifying microbial activity as a primary agent in the biodeterioration of historical artifacts [7,8]. Subsequent research has increasingly focused on the biodegradation of specific wooden cultural objects. For instance, Pan Jiao’s lab analyzed the microbial communities present on the lacquered surfaces of the Nanhai I shipwreck [9], while Hala A. M. Afif and colleagues investigated the mold infestation affecting the decorated, gilded wooden boards in the funerary complex of Sultan Al-Ashraf Qaitbay in Sudan [10].

Despite these advancements, targeted investigations concerning large-scale, above-ground wooden structures in northern China, particularly those constructed with traditional pine timber and protected using historic Tung oil finishing techniques, remain scarce. A notable research gap exists for heritage sites like Dajue Temple, where a well-established, specific conservation strategy to address such biodeterioration is currently lacking.

This study represents the first systematic investigation into the phenomenon of black staining on the wooden components of Dajue Temple [11,12]. Given that the conservation materials and techniques employed at Dajue Temple are consistent with those used in other major wooden architectural heritage sites, such as the Forbidden City, the findings from this research hold significant potential as a representative model for the conservation of similar heritage assets, offering broad applicability.

Consequently, this research aims to elucidate the origin of black discoloration on lacquered wooden surfaces of Dajue Temple and develop targeted prevention and control strategies. Its core objectives are: (1) to verify whether the staining stems from microorganisms; (2) upon confirmed microbial involvement, to characterize dominant microbial taxa, community structure and their wood degradation potential; and (3) to screen and develop safe and effective biocidal materials for the temple’s lacquered architectural components.

## 2. Materials and Methods

### 2.1. On-Site Investigation and Sample Collection

Sampling was conducted within areas of the Dajue Temple exhibiting deterioration of the lacquer-painted components. The sampling location, specific sampling points, ambient temperature, and relative humidity were meticulously documented. To minimize impact on the cultural relics, non-destructive or micro-destructive methods were employed for microbial sampling of the wooden artifacts. The procedures included: (1) Rapid assessment of microbial load in suspected areas using an ATP bioluminescence assay to preliminarily evaluate damage caused by microbial activity; (2) Swabbing of black-stained areas with sterile cotton swabs, followed by streak inoculation onto culture media to isolate primary pathogenic microorganisms using traditional cultivation techniques; (3) Additional sampling with sterile cotton swabs, which were immediately placed into centrifuge tubes containing sterile saline solution. All samples were transported to the laboratory under low-temperature conditions for subsequent molecular biological analyses.

### 2.2. Microbial Isolation, Cultivation, and Identification

#### 2.2.1. Microbial Isolation

Inoculated plates were transported to the laboratory and incubated at 28 °C in a constant-temperature incubator. After 3–5 days of cultivation, distinct colonies were selected based on morphological differences and subcultured onto fresh PDA medium (Solarbio Cat#P8931) for further purification. The growth status of colonies on streak plates was continuously observed and recorded until pure cultures were obtained.

#### 2.2.2. Single Colony Observation

Using the hyphal tip inoculation method, purified fungal isolates were inoculated onto the center of PDA solid medium plates and incubated at 28 °C. Colony growth was observed every 2–3 days, and the following morphological characteristics were documented: colony color, texture, hyphal density, margin morphology (regular or irregular), characteristics of aerial and substrate hyphae, and any color changes in the culture medium.

#### 2.2.3. Wet Chamber Experiment

Filter paper, a U-shaped glass rod, and glass slides were sequentially placed into a glass petri dish. The assembled chamber was sterilized by autoclaving at 121 °C for 30 min and then dried for subsequent use. In a laminar flow cabinet, molten PDA medium was poured into sterile glass petri dishes to form a thin layer. After cooling and solidification, small blocks (0.5–1 cm^2^) of the PDA medium were cut using a scalpel and placed at each end of the sterile glass slides within the chamber. A small amount of fungal spores was inoculated along the edges of the agar blocks using an inoculation loop, and a sterile coverslip was placed on top. Subsequently, 2 mL of 50% sterile glycerol was added to the filter paper to maintain humidity. The chambers were sealed, labeled, and incubated upright at 28 °C. Spore germination, hyphal growth, and sporulation were periodically observed. Images were finally captured using light microscopy.

#### 2.2.4. Sequencing of Purified Colonies

Mycelia were collected into 2 mL microcentrifuge tubes. Glass beads (24–48 mesh) and 500 μL of lysis buffer were added. Cells were thoroughly disrupted by vortexing for 7 min, followed by incubation in a 37 °C water bath for 30 min. Subsequently, 200 μL of 5 mol/L NaCl solution was added, mixed gently, and centrifuged at 13,000 rpm for 1 min. The supernatant was transferred to a new microcentrifuge tube. An equal volume of phenol/chloroform (1:1, *v*/*v*) was added to the supernatant for extraction, mixed gently by inversion, allowed to stand for 5 min, and centrifuged at 12,000 rpm for 3 min. The supernatant was collected, and this extraction step was repeated once. DNA was precipitated by adding two volumes of pre-chilled anhydrous ethanol and 0.1 volumes of 3 mol/L potassium acetate solution to the supernatant, followed by incubation at −20 °C for 30 min. The DNA precipitate was collected by centrifugation at 13,000 rpm for 10 min. The DNA pellet was washed twice with 400 μL of pre-chilled 70% ethanol, collected by centrifugation at 12,000 rpm for 5 min each time. After air-drying at room temperature, the DNA pellet was dissolved in 50 μL of ddH_2_O. DNA concentration and purity were assessed using a micro-spectrophotometer.

PCR amplification was performed in a 25 μL reaction volume containing 1 μL each of forward and reverse primers (ITS1: TCCGTAGGTGAACCTGCGG), 1 μL of template DNA, and 12.5 μL of premixed PCR mix (containing Taq polymerase, dNTPs, and buffer), with ddH_2_O added to reach the final volume. The PCR program consisted of initial denaturation at 98 °C for 3 min; followed by 33 cycles of denaturation at 98 °C for 10 s, annealing at 57 °C for 10 s, and extension at 72 °C for 50 s; a final extension at 72 °C for 5 min; and holding at 4 °C. Amplification products were verified by agarose gel electrophoresis. Following verification, PCR products were sent to Genewiz Biotechnology Co., Ltd. (Suzhou, Jiangsu, China) for sequencing using the ITS1 primer. The resulting sequences were compared against the NCBI database using BLAST+ 2.17.0 to determine the taxonomic identity of the isolates.

### 2.3. Qualitative Assessment of Lignin and Cellulose Degradation Ability

For cellulolytic activity assessment, fungi were cultured on CMC agar medium containing Congo red (Hopebio, Cat#HB8638, Qingdao, Shandong, China). Fungi capable of cellulose degradation produce clear zones (halos) around the colonies due to the decolorization of Congo red. Plates were placed on a white light transilluminator to observe and measure the hydrolysis zones.

For ligninolytic activity assessment, fungi were cultured on medium containing guaiacol (Solarbio Cat#M8320, Beijing, China). Fungal strains capable of lignin degradation were identified by their ability to grow on guaiacol plates and cause a color change in the medium (development of a reddish-brown color). The extent and intensity of the reddish-brown zone are indicative of the strain’s lignin-degrading potential.

### 2.4. Evaluation of Fungal Strains Susceptibility to Biocides

#### 2.4.1. Spore Preparation and Enumeration

Fungi were inoculated onto PDA solid medium and incubated at 28 °C for 7 days to obtain well-grown sporulating cultures. The plate surface was gently scraped with a sterile spreader to dislodge spores from hyphae. A 0.1% Tween 80 solution was added, and the plate was gently agitated to disperse the spores. The resulting spore suspension was transferred by pipette and filtered through 3–4 layers of sterile gauze to remove mycelial fragments, agar blocks, and debris. The filtrate was centrifuged, and the pellet was resuspended and diluted with sterile saline solution. Spore concentration was determined using a hemocytometer. The final spore suspension was adjusted to a concentration of 10^6^ spores per mL. This procedure was repeated for each fungal species used in the experiments. The obtained fungal spore suspensions were stored at 4 °C.

#### 2.4.2. Antifungal Inhibition Assay

Filter paper disks impregnated with the test antifungal agents were placed on the surface of solid medium previously inoculated with the pathogenic fungus. The antifungal agent diffuses into the agar, creating a concentration gradient. After incubation, the antifungal efficacy was evaluated by measuring the diameter of the inhibition zone formed around the disks. The specific procedure was as follows: A spore suspension was prepared as described in Section 2.4.1. Spores were evenly spread onto a PDA plate. Sterile filter paper disks (7 mm diameter) were placed onto the center of the inoculated plates. Each disk received 8 μL of the antifungal agent. Plates were incubated upright at 28 °C. The formation of inhibition zones was monitored periodically. The diameter of the inhibition zone is positively correlated with the sensitivity of the fungus to the tested antifungal agent. All experiments were performed independently in triplicate.

The antifungal agents used in this study are listed in Table 1 and Table 2. An antifungal agent was considered effective when the measured inhibition halo diameter was no less than 14 mm.

## 3. Results

### 3.1. In Situ Investigation and Confirmation of Microbial Activity

**Figure 1:**
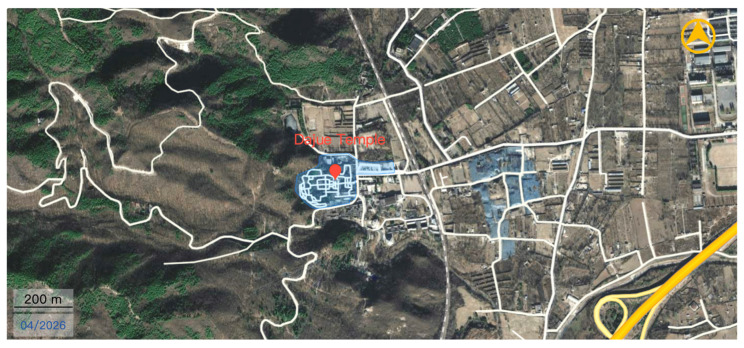
Satellite map showing the location and extent of Dajue Temple (from Amap).

**Figure 2:**
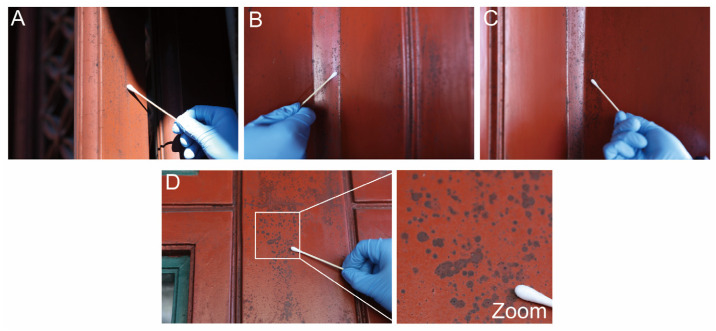
Sampling areas for microbiological analyses on the painted wooden component. (**A**) Main Hall, back door; (**B**) Qiyun Xuan, Left door frame; (**C**) Qiyun Xuan, Right door frame; (**D**) Qiyun Xuan, Right window frame.

The sampling area is the main hall, left and right door frames, and right window frame of Qiyun xuan in Dajue Temple, where black spot deterioration is severe; a total of 4 samples were collected, with 3 replicates for each sampling point. The sampling height is 1.5–2.0 m from the floor to avoid ground pollution and human touch (Figure 1 and Figure 2 and Table 3). The adenosine triphosphate (ATP) bioluminescence assay was employed to assess microbial activity on the stained wooden surfaces. ATP, a ubiquitous energy carrier in living organisms, serves as a reliable proxy for microbial biomass. ATP relative light unit (RLU) values measured at sites exhibiting black staining suggested 3917, 6022, 12,146, and 8572, strongly indicating that the formation of these stains is closely associated with microbial activity (Table 3).

### 3.2. Microbial Isolation, Purification, and Identification

**Figure 3:**
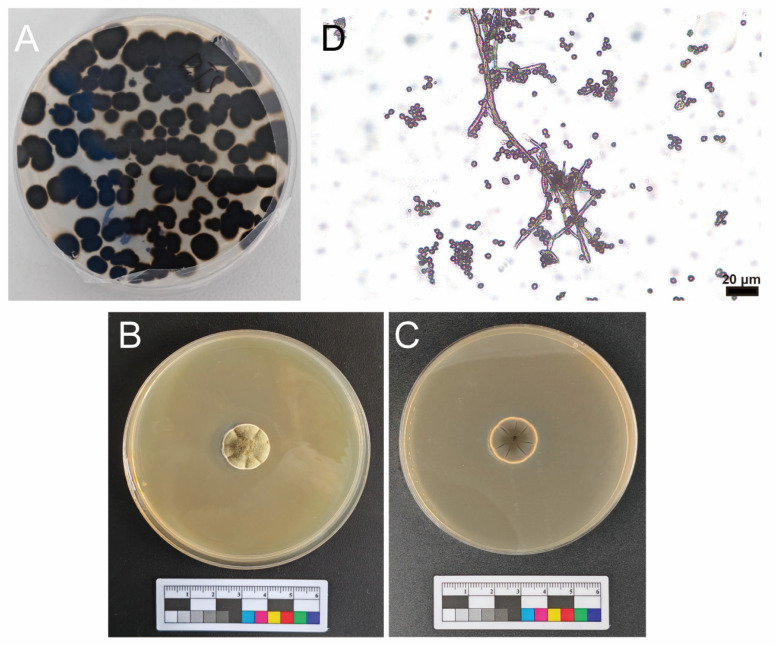
Morphological characteristics of the dominant fungus isolated from black-stained lacquered wooden components of Dajue Temple. (**A**) Colonial morphology of environmental isolates on PDA medium. (**B**) Obverse view and (**C**) reverse view of a purified colony cultivated on PDA medium. (**D**) Light micrograph of the fungal structures. Scale bar = 20 μm.

Laboratory cultivation of samples collected from the stained areas yielded predominantly pure cultures with no significant contamination (Figure 3A). The morphology of single colonies after two rounds of purification is shown in Figure 2B,C. The colonies were characterized by a raised, punctiform appearance on the obverse side, displaying a gray-green, velvety, floccose, or hairy texture (Figure 3B). The reverse side of the colonies exhibited a dark brown coloration and an umbonate shape with a distinct central depression, resembling a navel (Figure 3C). The isolated fungus was designated as strain DJSC.

Microscopic examination using slide culture techniques (Figure 3D) revealed that the conidiophores were distinctly differentiated, erect, and olive-brown in color. Conidiogenesis was sympodial, characterized by multiple apical branches forming successive whorls of ramoconidia, a pattern typical of the *Cladosporium* type. Conidia were deep olive-green, ellipsoidal to ovoid in shape, occasionally unicellular or multicellular, with discernible hila and scars. Integrating these morphological characteristics with molecular sequencing data, the dominant fungus was definitively identified as belonging to the genus *Cladosporium* spp. (Table 4).

### 3.3. Assessment of the Wood-Degrading Potential of the Isolated Cladosporium spp.

To evaluate the potential of the isolated *Cladosporium* spp. (strain DJSC) to degrade wood components, functional assays were performed using media supplemented with CMC, a cellulose analog, and guaiacol, an indicator of lignin modification.

**Figure 4:**
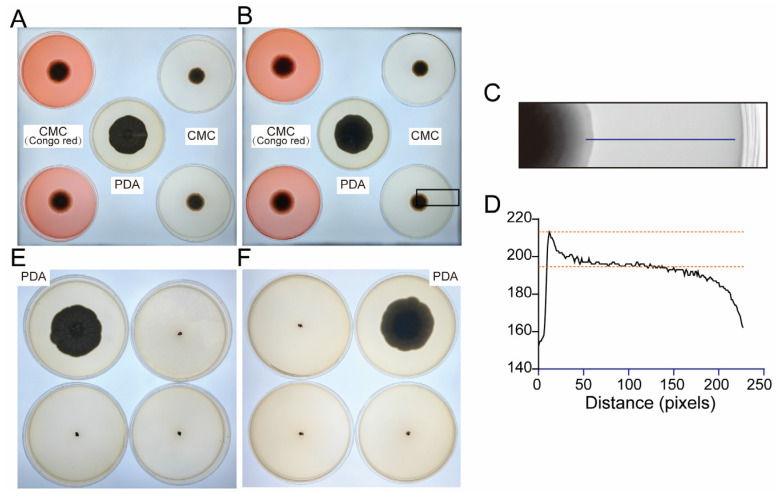
Degradation assays of strain DJSC on cellulose and guaiacol. Growth of DJSC on PDA (control), CMC, and CMC with Congo red media, (**A**) Plate obverse, (**B**) Plate reverse, (**C**) Gray value profile from colony center to plate edge with corresponding statistical analysis, (**D**) Growth of DJSC on PDA media with and without guaiacol, (**E**) Plate obverse, (**F**) Plate reverse.

On CMC agar plates stained with Congo red, distinct clear zones were observed surrounding the fungal colonies. ImageJ 1.54g analysis revealed a significant reduction in gray values from the colony margin to the periphery of the clear zone (Figure 4A), confirming the decomposition of the Congo red-cellulose complex. This result demonstrates the secretion of cellulases by strain DJSC, indicating its capability to degrade cellulose.

On PDA medium supplemented with guaiacol, the agar surrounding the colonies developed a light brown coloration (Figure 4B). This color change, indicative of guaiacol oxidation, suggests that the fungal strain possesses the potential enzymatic machinery for lignin transformation or degradation.

### 3.4. Biocide Susceptibility of Fungal Strains

**Figure 5:**
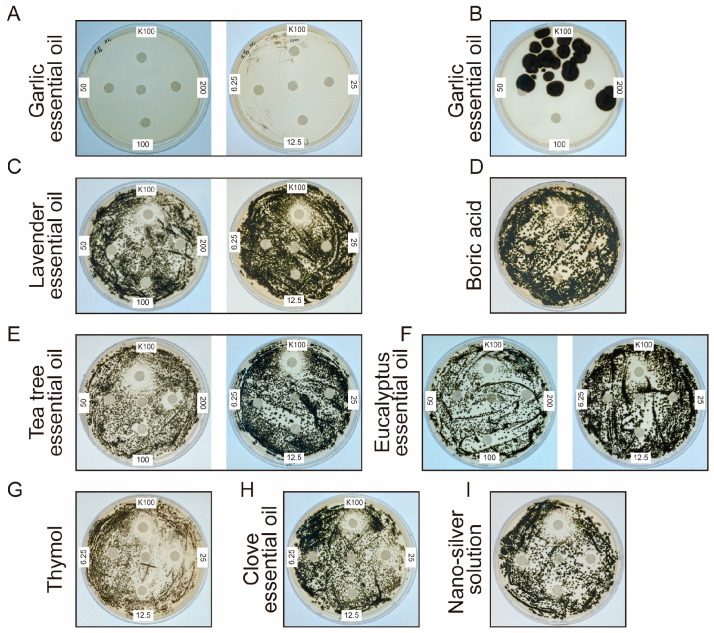
The inhibition of fungal growth by biocides. The status of *Cladospora* spp. after (**A**) adding garlic essential oil; (**B**) 12 days of incubation after adding garlic essential oil to the Petri dish and opening the lid for 8 days; (**C**) adding lavender essential oil; (**D**) adding boric acid; (**E**) adding tree essential oil; (**F**) adding eucalyptus essential oil; (**G**) adding thymol; (**H**) adding clove essential oil; (**I**) adding nano-silver solution.

To identify effective antifungal agents against DJSC, plates were inoculated with 10^6^ spores and incubated for four days. In the positive control group treated with K100 (Isothiazolinone), distinct inhibition zones formed around the filter paper discs. Conversely, on plates containing no antifungal agent, uniform fungal growth was observed across the entire agar surface, validating the experimental setup. Antifungal agents were tested at high (200, 100, 50 mg/mL) and low (25, 12.5, 6.25 mg/mL) concentration groups. As shown in Figure 5, plates treated with eucalyptus essential oil, lavender essential oil, low-concentration clove essential oil, nano-silver, and boric acid exhibited fungal growth covering the filter paper discs. Groups treated with 25 mg/mL thymol, 25 mg/mL clove essential oil, and 200 mg/mL and 100 mg/mL tea tree essential oil produced inhibition zones, albeit slightly smaller than those of the K100 positive control.

**Figure 6:**
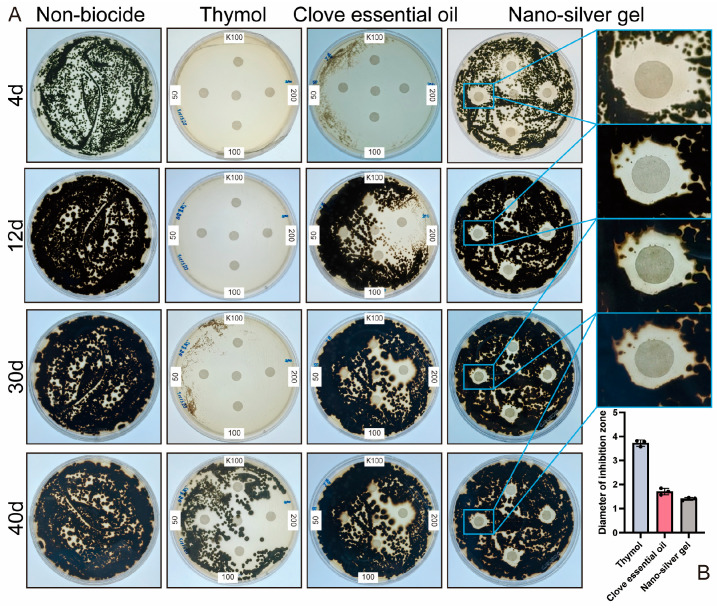
(**A**) The inhibition of fungal growth by three biocides; (**B**) Diameter of inhibition zone (cm) of thymol (200 mg/mL), clove essential oil (200 mg/mL) and nano-silver gel on day 40. The black dots represent the diameters of inhibition zones from three independent measurements.

Results from Figure 5 and Figure 6 indicated that, within the tested concentration range, antifungal efficacy was generally concentration-dependent, with higher concentrations yielding stronger effects. Five antifungal treatments demonstrated particularly pronounced activity. Specifically, high-concentration thymol, high-concentration clove essential oil, and both high and low concentrations of garlic essential oil resulted in over 80% of the plate area being free of visible fungal growth on day 4. And after 40 days, the diameter of the inhibition zone of thymol, clove essential oil and nano-silver gel could still reach 3.8, 1.8, and 1.4 cm, respectively. Additionally, the area covered by silver ion gel also showed no apparent colony formation. Based on these initial efficacy results, the antifungal agents were preliminarily ranked as follows: garlic essential oil > thymol > clove essential oil > silver ion gel > other tested agents.

## 4. Discussion

Dajue Temple, with its origins tracing back to the Liao Dynasty (founded in 1068 AD), has endured for over a millennium through successive imperial reigns, embodying profound historical and cultural significance. Designated a National Key Cultural Relics Protection Unit by the State Council in May 2006, its preservation status and conservation practices represent a critical area of inquiry within the field of cultural heritage, particularly as a prominent example of above-ground wooden architectural heritage.

However, during routine maintenance, conservators at Dajue Temple observed extensive black staining on the surfaces of painted wooden components. This phenomenon not only compromises the esthetic integrity of the relics but, more critically, signals an underlying risk of lacquer exfoliation and wood deterioration, thereby posing a direct threat to the structural safety of the artifacts themselves. In February 2025, we conducted an on-site investigation of the affected painted components, focusing on key architectural elements such as wooden columns and door and window frames within the main halls. Our survey confirmed the widespread presence of these black stains. Subsequent ATP bioluminescence assays [13] performed on the stained areas provided definitive evidence that this discoloration is attributable to microbial infestation.

A robust body of research has established microbial infestation as a core and long-term deterioration driver for above-ground wooden cultural heritage [14]. Such artifacts, chronically exposed to fluctuating environmental conditions, present a complex substrate comprising wood and surface coatings that readily support microbial colonization and proliferation, leading to composite material degradation [5,11,,15,16,,17]. Consequently, long-term monitoring and targeted microbial control should be regarded as fundamental components of any comprehensive conservation strategy for these structures. In this context, the present study focused on the black stain biodeterioration affecting the painted components of Dajue Temple. By centering on the dominant fungal populations, we systematically investigated their biodegradative mechanisms, screened for safe and effective antifungal agents, and compiled foundational data. This work aims to provide both a theoretical framework and practical technical references for the future prevention and control of fungal deterioration in comparable lacquered-pine composite components found in historic above-ground wooden architecture.

To precisely characterize the microbial agents responsible for the observed staining, we cultured samples collected during the on-site investigation, followed by purification, morphological characterization, and Sanger sequencing. This integrated approach unequivocally identified the dominant fungus as belonging to the genus *Cladosporium* spp.

*Cladosporium*, a large genus within the Ascomycota (class *Dothideomycetes*), is ubiquitously distributed in both outdoor and indoor environments and is frequently isolated from organic substrates such as wood, paint, and paper. Its metabolic processes often involve the production of pigments, resulting in the formation of characteristic black or dark brown colonies on host surfaces [18,19,20,21]. This feature is highly consistent with the macroscopic appearance of the black stains observed on the painted components of Dajue Temple. Ecologically, *Cladosporium* species frequently function as successors and accelerators in the later stages of wood decay, following the initial degradation facilitated by primary wood-rotting fungi. Furthermore, members of this genus are recognized as potential allergens, capable of inducing respiratory issues such as allergies and asthma in humans, including visitors and site personnel [22]. Therefore, targeted management of *Cladosporium* colonization on the painted surfaces of Dajue Temple is imperative, both for artifact preservation and for public health considerations.

As the most efficient decomposers of wood in many ecosystems, the potential of fungi to degrade their principal components directly determines the magnitude of the threat they pose to wooden artifacts. In this study, we systematically analyzed the cellulolytic and ligninolytic potential of the isolated *Cladosporium* strain. Our experimental results demonstrate that this strain is not only capable of stable survival on the surface of the painted wooden components but also possesses the metabolic capacity to secrete key enzymes, including cellulases and lignin-modifying enzymes, facilitating the gradual degradation of cellulose and lignin within the wood structure. Cellulose and lignin constitute the fundamental structural framework of wood. Their degradation directly compromises the integrity of wood cell walls, leading to structural loosening, loss of mechanical strength, cracking, and advanced decay. Moreover, this process undermines the adhesion between the lacquer layer and the wooden substrate, accelerating lacquer exfoliation. Cumulatively, these effects pose a substantive structural threat to the integrity of the painted wooden elements at Dajue Temple.

Several factors may contribute to the proliferation of *Cladosporium* on these surfaces. First, in addition to the organic polymers of the wood itself—cellulose, hemicellulose, and lignin—which provide a foundational carbon source, the historic timber, having endured for centuries, may contain accumulated inorganic ions that could promote microbial growth and metabolism. Second, the aging and degradation of the lacquer coating may release organic compounds that further stimulate fungal metabolic activity [23]. However, the specific validity and relative contributions of these hypothesized triggers require further investigation.

Prior to this study, site conservators had attempted routine cleaning methods, including wiping with water and applying ethanol, to address the black staining. These approaches proved suboptimal. Water-based cleaning offered only transient removal of surface discoloration, with the problem recurring rapidly. Ethanol treatment, while operationally simple, is generally inadvisable for lacquered wood. Its high volatility results in poor persistence, and it carries the risk of solubilizing or damaging the aged lacquer film, potentially exacerbating wood desiccation and cracking, while also introducing safety hazards. These limitations underscore the critical need for safe, efficient, and environmentally benign antifungal agents with long-term efficacy suitable for application on lacquered wooden artifacts.

In response, this study systematically screened a range of antifungal substances against the isolated *Cladosporium* strain [24]. Garlic essential oil exhibited high volatility. Upon opening the culture plates, fungal colonization by DJSC rapidly progressed across the entire agar surface, regardless of whether garlic oil was reapplied to the filter paper disks (Figure 5B). Furthermore, garlic essential oil demonstrated poor stability, being sensitive to light, heat, and alkaline conditions, and possessed a strong, pungent odor characteristic of garlic. These properties render it unsuitable for treating cultural heritage artifacts or for use in open display environments. Additionally, the biocide K100 was deemed impractical for large-scale application due to its high cost. Consequently, our results demonstrate that thymol, clove essential oil, and silver ion gel each exhibited significant antifungal activity under the experimental conditions employed. These agents are particularly attractive due to their natural origins, relatively low toxicity profiles, and potential for controlled-release formulations.

Nevertheless, it is imperative to acknowledge that cultural heritage artifacts possess irreplaceable value. Consequently, any biocidal agent must undergo comprehensive evaluation for material compatibility, long-term safety, and potential environmental impact prior to field application. While the antifungal agents identified in this study demonstrate promising short-term control of microbial growth in vitro, further research is essential to assess their long-term effects (including compatibility and aging effects) on the historic materials. This includes rigorous testing to ensure that their application does not inadvertently introduce new pathways for physicochemical deterioration, thereby ensuring that conservation interventions are both effective and sustainable in the long-term preservation of this invaluable heritage site.

## 5. Conclusions

This study demonstrates definitively that the extensive black staining observed on the painted wooden components of Dajue Temple is attributable to fungal activity, with *Cladosporium* spp. identified as the predominant causative agent. Through integrated on-site investigation, laboratory cultivation, morphological and molecular characterization, and functional assays, we establish that this fungus possesses the metabolic capacity to degrade both lignin and cellulose, thereby posing a substantive threat to the long-term structural integrity of these historic painted surfaces. Furthermore, this work has identified several promising antifungal candidates—thymol, clove essential oil, and silver ion gel—that exhibit potent inhibitory effects against the isolated *Cladosporium* strain. Collectively, these findings not only elucidate the etiology of a critical deterioration phenomenon at a major heritage site but also provide a foundational framework for developing targeted, evidence-based conservation strategies applicable to Dajue Temple and analogous lacquered wooden architectural heritage.

## Figures and Tables

**Table 1:** Antifungal agents used in this study.

Fungicide	Source	Main Fungicidal Components	Concentration (mg/mL)
K100 (isothiazolinone)	Euxyl^®^ K100, Norderstedt, Germany	0.75%	0.5%
Nano-silver gel	Sliver Care, Shenzhen, China	Nano-silver	Ag ion: 750–1050 μg/g
Nano-silver solution	YI JIE SHI, Sichuan, China	Nano-silver	Ag ion: 50–100 mg/L
Eucalyptus essential oil	Vita, Shanghai, China	1,8-Clineole (Eucalyptol)	200, 100, 50, 25, 12.5, 6.25
Clove essential oil	Vita, Shanghai, China	Eugenol	200, 100, 50, 25, 12.5, 6.25
Tea tree essential oil	Vita, Shanghai, China	Terpinen-4-ol	200, 100, 50, 25, 12.5, 6.25
Lavender essential oil	Vita, Shanghai, China	Linalool, Linalyl acetate	200, 100, 50, 25, 12.5, 6.25
Garlic essential oil	Vita, Shanghai, China	Sulfur compounds	200, 100, 50, 25, 12.5, 6.25
Boric acid solution	Ounuokang, Quanzhou, China (CAS: 10043-35-3)	H_3_BO_3_	3% ± 0.3% (*w*/*w*)
Thymol	MACKLIN, Shanghai, China (CAS: 89-83-8)	C_10_H_14_O	200, 100, 50, 25, 12.5, 6.25

**Table 2:** Analysis of chemical composition of the essential oil (data from Shanghai Vita Co., Ltd., Shanghai, China).

Essential Oil	Components	Relative Density (25/25 °C)	Refractive Index
Eucalyptus essential oil	1,8-Cineole (Eucalyptol), 80.36%	0.913	1.4626
Clove essential oil	Eugenol, 85.12%	1.049	1.530
Tea tree essential oil	1,8-Cineole, 4.81% Terpinen-4-ol, 41.21%	0.891	1.479
Lavender essential oil	Camphor, 0.24% Linalool, 39.75% Linalyl acetate, 27.74% Lavandulyl acetate, 7.39%	0.880	1.460
Garlic essential oil	Allicin, 30.3%	1.072	1.579

**Table 3:** Sampling locations, specific sampling points, and ATP content at Dajue Temple.

NO.	Building Name	Specific Sampling Point	ATP Content (RLU)
A	Main Hall	Back door, north side	3917
B	Qiyun Xuan	Left door frame	6022
C	Qiyun Xuan	Right door frame	12,146
D	Qiyun Xuan	Right window frame	8572

**Table 4:** Molecular identification of pure strains isolated from lacquerware surfaces.

Taxonomy	Closet Strain	Similarity (%)	Accession Number (GenBank Database)	GeneBank Number
DJSC	*Clad* *osporium*	100%	AB572909.1	PZ043134

## Data Availability

The original contributions presented in this study are included in the article. Further inquiries can be directed to the corresponding authors.

## Abbreviations

The following abbreviations are used in this manuscript:

PDA	Potato Dextrose Agar
CMC	Carboxymethylcellulose
